# Five years of post-validation surveillance of lymphatic filariasis in Thailand

**DOI:** 10.1186/s40249-023-01158-0

**Published:** 2023-12-06

**Authors:** Prapapan Meetham, Rawadee Kumlert, Deyer Gopinath, Siriporn Yongchaitrakul, Tanaporn Tootong, Sunsanee Rojanapanus, Chantana Padungtod

**Affiliations:** 1 Division of Vector Borne Diseases, Department of Disease Control, Ministry of Public Health, Nonthaburi, Thailand; 2 World Health Organization (WHO), Country Office, Nonthaburi, Thailand; 3 Department of Disease Control, Ministry of Public Health, Nonthaburi, Thailand

**Keywords:** Lymphatic filariasis, Elimination, Post validation, Surveillance, Neglected tropical diseases, Thailand

## Abstract

**Background:**

The World Health Organization (WHO) validated Thailand in 2017 as having eliminated lymphatic filariasis (LF) as a public health problem with recommendations for continued surveillance. This article describes measures and progress made in Thailand with post-validation surveillance (PVS) of LF from 2018 until 2022.

**Methods:**

The implementation unit (IU) is a sub-village in 11 former LF endemic provinces. Human blood surveys are targeted in 10% of IUs each year. In *Wuchereria bancrofti* areas, filaria antigen test strips (FTS) are used, and in *Brugia malayi* areas, antibody test kits (Filaria DIAG RAPID) are used. Positive cases are confirmed by thick blood film (TBF) and polymerase chain reaction (PCR). Vector surveys for mosquito species identification and dissection for microfilaria (Mf)/filarial larvae are done in 1% of IUs where human blood surveys are conducted. Human blood surveys using FTS are conducted among migrants in five provinces. Surveillance of cats is done in areas that previously recorded > 1.0% Mf rate among cats. Morbidity management and disability prevention (MMDP) are done every 2 years in LF-endemic areas where chronic disease patients reside.

**Results:**

From 2018 to 2022, in a total of 357 IUs in 11 provinces, human blood surveys were conducted in 145 IUs (41%) with an average population coverage of 81%. A total of 22,468 FTS and 27,741 FilariaDIAG RAPID were performed. 27 cases were detected: 3 cases of *W. bancrofti* in Kanchanaburi province and 24 cases of *B. malayi* in Narathiwat province. 4 cases of *W. bancrofti* were detected in two provinces through routine public health surveillance. Vector surveys in 47 IUs detected *B. malayi* Mf filarial larvae only in Narathiwat province. Chronic LF patients reduced from 114 in 2017 to 76 in 2022. Surveys among 7633 unregistered migrants yielded 12 cases of *W. bancrofti*. Mf rate among cats in Narathiwat province declined from 1.9% in 2018 to 0.7% in 2022. MMDP assessments revealed gaps in healthcare provider’s management of chronic cases due to staff turnover.

**Conclusions:**

In 2022, after 5 years of PVS, Thailand re-surveyed 41% of its previously endemic IUs and demonstrated ongoing transmission in only one province of Narathiwat, where Mf prevalence is below the WHO provisional transmission threshold of 1%. This study highlights the importance of continued disease surveillance measures and vigilance among health care providers in LF receptive areas.

**Graphical Abstract:**

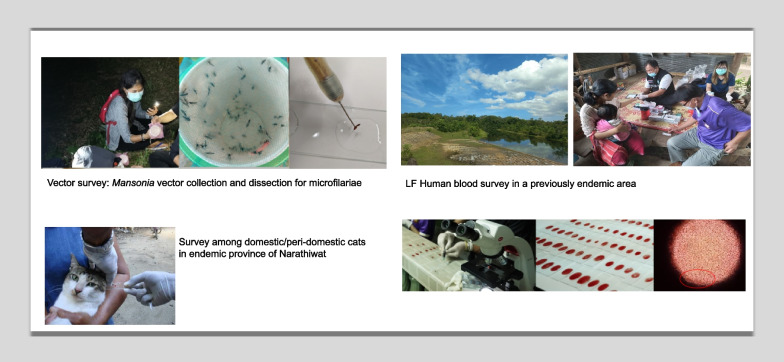

## Background

### Lymphatic filariasis (LF) epidemiology in Thailand

*Wuchereria bancrofti* endemic provinces are located in the north and central Thailand, and *Brugia malayi* endemic provinces are in the south Thailand. Seven provinces endemic for *W. bancrofti*, transmitted by *Aedes niveus* mosquito species; and four provinces endemic for *B. malayi*, transmitted by *Mansonia* mosquito species [1–3]. Annual mass drug administration (MDA) with diethylcarbamazine citrate (DEC) and albendazole (ALB) was implemented in a total of 357 implementation units (IUs) in these 11 LF endemic provinces (total population of the 357 sub-villages in 2002 was 124,496) for a total of 5 rounds over five-year period (2002–2006). The IU was a sub-village. Although all 11 provinces are endemic, 4 of these provinces—Mae Hong Son, Tak, Kanchanaburi, and Narathiwat, accounted for 336 of 357 (94%) endemic sub-villages. Additional annual rounds of MDA were required in 87 IUs of Narathiwat province from 2007 to 2011 due to persistent infection. Three transmission assessment surveys (TAS-1, TAS-2, and TAS-3) were conducted over 2012–2017, where all 357 IUs were surveyed and indicated that transmission was below the TAS critical cut-off threshold in all five evaluation units (EUs). Contact tracing of all Mf cases in all three TAS yielded no positive cases [3].

A 2001 survey of the chronic disease burden for lymphatic filariasis established a register of people in endemic provinces with lymphedema/elephantiasis. The number of persons declined from 284 in 2001 to 99 patients in 2017 who are followed-up under 34 health centers, of which a total of 69 patients (70%) were under the care of 14 health centers in just one province of Nakhon Si Thammarat [3].

Since 2001, the Thai Ministry of Public Health (MOPH) set up the migrant health insurance scheme for all migrants (registered and unregistered) who are not covered by social health insurance, allowing mandatory health screening (during the first entry and subsequent yearly renewal of the residence permit) [4] which includes testing for *bancroftian* microfilaria (Mf provocation test with DEC) done at all district hospitals and for which a full course of treatment (single dose of DEC + ALB) is offered if found to be positive. Results are published elsewhere [3].

In Narathiwat Province, surveys and treatment with ivermectin among domestic cats commenced in 1994 and were conducted annually in that province as a measure to prevent possible zoonotic transmission. In areas with > 1.0% Mf rate among cats in Narathiwat province, annual ivermectin treatment resulted in a decline in Mf prevalence among cats from 8.07% in 1995 to as low as 0.84% in 2015 [3].

### LF program structure in Thailand

**Fig. 1:**
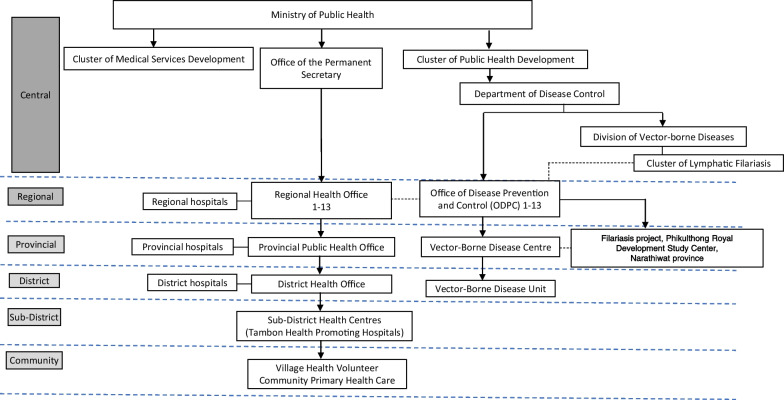
Structure of NPELF of Thailand: as of 2020, there were 1367 public and private medical facilities, 9759 Sub-District Health Centres and 1.04 million village health volunteers. *NPELF* National Programme to Eliminate LF

In 1961, the Division of Lymphatic Filariasis was established under the Department of Health with a primary strategy of using DEC to control LF in known endemic areas. In 2001 the National Programme to Eliminate LF (NPELF) was launched in Thailand [5]. The structure and organization of the program are shown in Fig. 1. Roles and responsibilities are described elsewhere [3]. The Royal Thai Government has also ensured that resources are allocated for LF surveys, integrated vector control efforts, and screening among at-risk groups. With the establishment of the UHC scheme in 2001 and subsequently migrant health insurance schemes, the provision of free morbidity management and disability prevention services were extended to the sub-district Tambon Health Promotion Hospital and for both registered and unregistered migrants. The Thai Royal Filaria Project established the Phikulthong Royal Development Study Center in Narathiwat Province and continues to provide all necessary support with infrastructure and required personnel for LF post-validation efforts in Narathiwat Province.

### Lymphatic filariasis post validation surveillance (PVS) in Thailand (2018–2027)

**Fig. 2:**
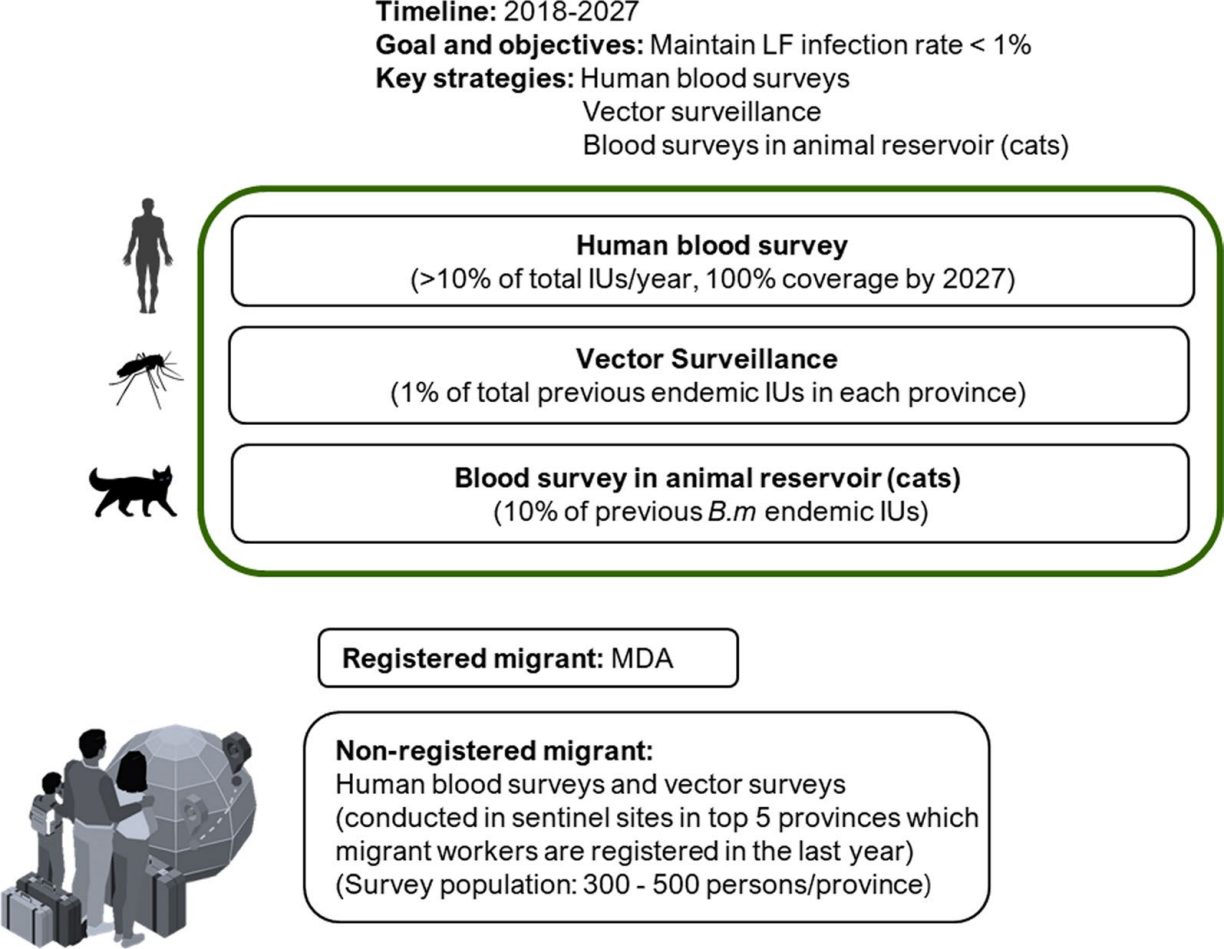
National strategic plan for Lymphatic Filariasis (LF) Post validation surveillance (PVS) in Thailand. *IU* implementing unit, *MDA* mass drug administration

DVBD has developed national guidelines for PVS of LF over a provisional 10-year post-validation (2018–2027) period. Although guidance on PVS from WHO is pending, proposed provisional guidance from WHO notes that PVS activities should be conducted for at least 10 years [6]. As a primary and minimum aim, current recommendations are to ensure that recrudescence hasn’t occurred; infection in evaluation units (EUs) is still below target thresholds. The secondary and advanced aim is to verify the elimination of transmission, criteria for which are yet to be identified. Key PVS strategies in Thailand include human blood surveys in sub-villages and among migrant populations, vector surveillance, blood surveys in animal reservoirs (cats), and morbidity management and disability prevention (MMDP). This is summarized in Fig. 2.

## Methods

### Human blood surveys

**Fig. 3:**
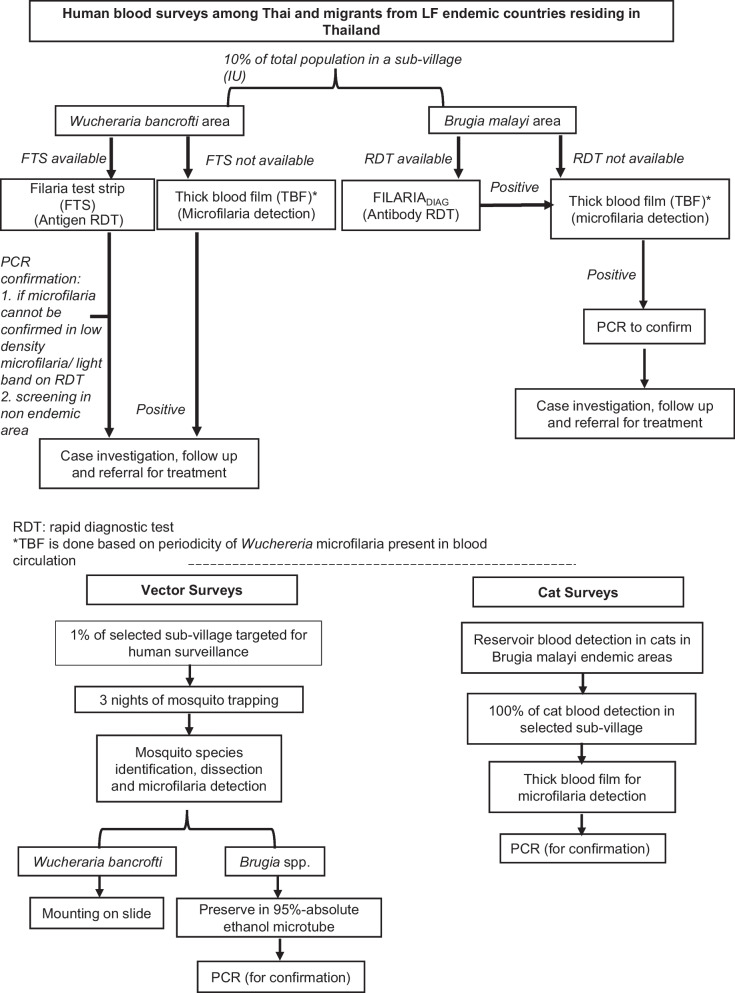
LF post validation surveillance measures in former endemic areas: human, vector and cat surveys. *LF* Lymphatic filarioasis, *FTS* Filaria antigen test strip

Target areas for human blood and vector surveys are previous endemic areas (11 provinces, 357 IUs) based on clinical case/Mf prevalence, vector density, and environmental factors favorable for vector breeding. Human blood surveys are conducted in 10% of sub-villages each year and are driven by the provincial vector-borne disease control (VBDC) and regional level office of disease prevention and control (ODPC) staff during the daytime using FTS in *W. bancrofti* areas and antibody test kits (FilariaDIAG RAPID) *in B. malayi* areas as shown in Fig. 3. TBF is usually taken after 6:00 PM based on the nocturnal sub-periodicity of *W. bancrofti* and *nocturnal* periodicity of *B. malayi*. TBF are stained with Giemsa. PCR is done at DVBD for confirmation if microfilaria cannot be determined by FTS (light/faint band) or if positive cases by FTS are detected in a non-endemic LF area [7]. All *B. malayi* antibody-positive cases are confirmed by PCR. All positive cases are treated with DEC. For *W. bancrofti* infection, DEC 6 mg/kg single dose is given every 6 months for 2 years. For *B. malayi* infection, DEC 6 mg/kg for 6 days repeated every 6 months for 2 years. Treatment of family members or co-travellers are based on the case investigation findings, a standardized LF case investigation form of the national disease surveillance guidelines [8]. Targeted drug administration/mass drug administration will be considered based on the case investigation of new case/s, for example, where there are new indigenous case/s reported versus imported cases or if the prevalence rate is > 1%. The objective of human blood surveys will be to achieve geographical coverage of 100% in previously endemic areas within 10 years of the PVS phase.

### Vector surveys

Vector surveys are conducted by VBDC and ODPC staff in 1% of sub-village where human blood surveys are conducted that year. Selection is guided by sub-villages where the current LF vectors are predominant (*B. malayi* areas). Three nights of mosquito trapping are done, usually from 6:00 PM to 8:00 PM. where mosquito species identification and Mf and larval stage detection are done. Procedures for collection, identification, and dissection follow the DVBD entomology guidelines. *Brugia* spp. are sent for PCR confirmation at DVBD [7].

### Surveys among registered and unregistered migrant workers

Surveys among registered and unregistered migrant workers are done similarly before validation, where surveys are conducted in two ways. The first are annual LF surveys initiated by the VBDC and VBDU in five provinces where there are a high number of registered migrant workers. These surveys are supervised by the DVBD. The PVS target is to screen 300–500 unregistered migrants in these areas, commonly in construction sites, plantations, and factories. The second form of surveillance is through health check-ups conducted by various agencies, including the Provincial Health Office, the Division of International Disease Control Ports, the Department of Medical Service of the Bangkok Metropolitan Authority (BMA), and the One Stop Service (OSS), Office of the Permanent Secretary of MoPH.

### Surveys among persons in displaced population camps

There are 9 displaced population (DP) camps or temporary shelters located in 4 provinces in Thailand along the border with Myanmar, which have existed for 10 to 20 years. These camps are under the responsibility of the Ministry of Interior and Health services are managed by an international NGO. Health surveillance data are reported to the provincial health office. The current population is estimated at 84,133. Data on LF surveys and cases are not available before the start of the PVS. In 2019, LF surveys were conducted in all 9 camps. The screening was based on available financial, human resources, and availability of FTS. If a case is confirmed during human blood surveys, a case investigation will follow, and treatment will be given regardless of nationality or legal status.

### Cat surveys and treatment to prevent possible zoonotic transmission of LF

In the four provinces previously endemic for *B. malayi*, active surveillance of cats in areas that previously recorded > 1.0% Mf rate among cats, using thick blood film (TBF), is done along with treatment for cats found to be Mf positive from TBF. Surveys are done in the same village as the human surveys for the year and are conducted by staff of the Thai Royal Filaria Project in Narathiwat, VBDC, and VBDU staff. Surveys are conducted throughout the day. The ear lobe of cats is pricked to collect 6 drops (30 µl) of blood and examined by TBF. Cats positive for Mf are given ivermectin at the dosage of 0.1 ml/kg delivered subcutaneously. A cat household registration form has been established, which contains information on household numbers, cat/s—characteristics, weight, age and sex, TBF results, parasite species, etc. Positive TBF is confirmed with PCR at DVBD [7]. In practice, during surveys, all cats surveyed (tested) are treated at the same time due to difficulty in following up.

### LF chronic disease survey and management

LF chronic disease survey and management includes MMDP and health facility assessments in LF endemic areas where chronic disease patients reside under the care of a sub-district health facility in each province. The register of chronic patients is updated every year by the province and DVBD. The primary tools are the MMDP kit for chronic patients and the WHO Direct Inspection Protocol version 1.1 for health facilities. Home visits to patients and trainings for health facility staff are also included. These activities are conducted every 2 years. The MMDP strategy driven by the sub-district health facility staff is patient-centered, with services given in a comprehensive care package closest to the patient’s home aligned to the WHO guidelines [9, 10].

### Health promotion

Health education on LF is done simultaneously during all PVS activities. Regional offices (ODPC) and primary health care facilities also include LF education during general health education activities throughout the year in LF endemic areas. DVBD, as part of health education for vector-borne disease, also transmits specific LF prevention messaging through online social media channels and distribution of printed materials. Social media targets broader coverage nationwide, while specific education activities and materials are targeted to the 11 LF endemic provinces and family members/caregivers of LF patients as part of the MMDP during home visits.

### Routine health surveillance

Routine health surveillance is done through all health facilities and includes all diseases. A suspected case of LF is further investigated, tested (TBF or FTS or FilariaDIAG RAPID), and if positive, reported through routine health surveillance. LF is a notifiable disease in Thailand.

### Data analysis

All raw data except for the LF chronic disease survey, were obtained from the DVBD LF surveillance program and entered in Microsoft Excel 2007 worksheet. The chronic disease survey questionnaire was translated from Thai to English by professional translators with experience in the medical domain. Descriptive statistics were performed by calculating frequency (number and percentage) and mean for categorical and numeric variables, respectively.

## Results

### Human blood surveys

The cumulative coverage and results of surveys in the targeted 11 provinces over 2018–2022 is summarised in Table 1. “Target population” refers to the population targeted in each sub-village every year of the survey. Over 2018–2022, coverage of IUs was 100% in the provinces of Chiang Mai, Krabi, and Nakhon Si Thammarat, where there were three or fewer IUs in each of these provinces. Although all IUs were surveyed in these provinces, population coverage in the surveys varied, with a median of 97.2% in Chiang Mai, followed by 63.2% in Krabi and 50.1% in Nakhon Si Thammarat. No cases were detected in these provinces through FTS over 2018–2022. In the provinces that border neighboring Myanmar—Mae Hong Son (76 IUs), Tak (124 IUs), Kanchanaburi (49 IUs), Ratchaburi (4 IUs), and Ranong (2 IUs), only Kanchanaburi province reported 3 positive cases of *W. bancrofti* by FTS in 2021. The IU coverage in Kanchanaburi over 2018–2022 was 38.8%, and the median population coverage was 74.2%.

**Table 1:** Results of human blood surveys in 11 provinces: PVS 2018–2022

Province (total No. of IUs)	Year^a^	No. of IUs surveyed	Population in surveyed IUs	No. tested with FTS	No. of Ag+ cases	No. tested with TBF	No. of Mf+ cases	AGR (%)	Cumulative Mf rate (%) and [range]	Population coverage in surveyed IUs (%)
Mae Hong Son (76)	2019	8	1143	909	0	0	0	0	0	79.52
	2021	8	1895	1411	0	0	0	0	0	74.45
	2022	9	1763	1159	0	0	0	0	0	65.74
Chiang Mai (2)	2021	1	250	236	0	0	0	0	0	94.40
	2022	2	211	211	0	0	0	0	0	100.00
Tak (124)	2018	11	2587	0	0	1852	0	0	0	71.58
	2019	18	4878	4461	0	0	0	0	0	91.45
	2020	12	4144	3128	0	0	0	0	0	75.48
Kanchanaburi (49)	2019	5	3411	3063	0	0	0	0	0	89.79
	2020	3	1395	1275	0	0	0	0	0	91.39
	2021	6	1409	1305	3	0	0	1.25	0	92.61
	2022	5	1931	1657	0	0	0	0	0	85.81
Lamphun (3)	2022	1	238	238	0	0	0	0	0	100.00
Ratchaburi (4)	2019	1	993	824	0	0	0	0	0	82.98
	2020	2	522	483	0	0	0	0	0	92.52
Ranong (2)	2020	1	1692	510	0	0	0	0	0	30.14
	2021	1	1458	1080	0	0	0	0	0	74.07
	2022	1	352	300	0	52	0	0	0	100.00
Krabi (2)	2019	1	799	0	0	193	0	0	0	24.15
	2020	1	131	0	0	131	0	0	0	100.00
Nakhon Si Thammarat (2)	2018	1	1388	0	0	530	0	0	0	38.18
	2020	2	917	0	0	372	0	0	0	40.56
	2021	1	132	0	0	132	0	0	0	100.00
Surat (6)	2018	1	502	0	0	487	0	0	0	97.01
	2020	2	845	0	0	666	0	0	0	78.81
	2021	1	116	116	0	0	0	0	0	100.00
	2022	1	102	102	0	0	0	0	0	100.00
Narathiwat (87)	2018	9	6115	0	0	4911	9	0	0.18 [0–0.77]	80.31
	2019	10	6847	0	0	6284	8	0	0.12 [0–3.03]^b^	91.77
	2020	8	5200	0	0	3791	4	0	0.10 [0–0.45]	72.90
	2021	8	4819	0	0	3989	1	0	0.02 [0–0.42]	82.77
	2022	11	5292	0	0	4351	2	0	0.04 [0–0.53]	82.21

*Mf+* microfilaria positive, *AGR* antigen rate, *IU* implementation unit, *FTS* Filaria antigen test strip, *TBF* Thick blood film,* PVS* Post-validation surveillance

^a^Only years where surveys were conducted are tabled. No surveys were conducted in years not shown

^b^ Mf rate of 3.03 was recorded in Paye IU with 2 positive cases among 66 persons tested by TBF

In the southern provinces of Surat Thani (6 IUs) and Narathiwat (87 IUs), the coverage of IUs in Surat Thani was 66.7% and 51.7% in Narathiwat. The median population coverage in Surat Thani was 100%, and 88.7% in Narathiwat.

In Narathiwat province, a total of 45 out of a total of 87Us were surveyed over 2018–2022. TBF detected 24 Mf positive cases in 4 subdistricts affecting 10 IUs, Table 2*.* In all IUs in Narathiwat province, the microfilaria rate was < 1% during each survey except for one IU, Paye, in 2019, with 2 cases and Mf rate of 3.0% (population surveyed was 66).

**Table 2:** Mf positive cases in Narathiwat province: 2018–2022

Year	Province	District	Sub-district	Sub-village	Target population	TBF	Population coverage (%)	Mf+	Mf rate (%)
2018	Narathiwat	Mueang	Kaluwo	Koksila	656	440	67.1	1	0.2
2018	Narathiwat	Tak Bai	Bang Khun Thong	Bangkhunthong	1411	1252	88.7	1	0.1
2018	Narathiwat	Su-ngai Kolok	Puyo	Towo-og	960	775	80.7	4	0.5
2018	Narathiwat	Su-ngai Padi	Su-ngai Padi	Tasaenuea	462	389	84.2	3	0.8
2019	Narathiwat	Su-ngai Kolok	Puyo	Gubae-e-gae	595	595	100.0	5	0.8
2019	Narathiwat	Su-ngai Padi	Su-ngai Padi	Pawei	223	223	100.0	1	0.4
2019	Narathiwat	Su-ngai Padi	Su-ngai Padi	Paye	67	66	98.5	2	3.0
2020	Narathiwat	Tak Bai	Phron	Banyai	485	441	90.9	2	0.5
2020	Narathiwat	Su-ngai Kolok	Puyo	Gubae-e-gae*	595	595	100.0	2	0.3
2021	Narathiwat	Su-ngai Padi	Su-ngai Padi	Banta	248	239	96.4	1	0.4
2022	Narathiwat	Tak Bai	Bang Khun Thong	Kok-ngu	377	374	99.2	2	0.5
					6079	4796	90.6	24	0.7

*TBF* thick blood film

^a^In this sub-village population coverage was achieved in 2019 (100%). In 2020, a repeat survey was done for research purposes and detected 2 additional Mf+ cases

### Routine public health surveillance

In 2022, in Ratchaburi province, 2 cases were detected through passive/routine case surveillance in a new IU. One case was diagnosed as Mf positive, and another case was Lymphoedema. In Narathiwat in 2022, 2 cases were detected through routine surveillance. Both cases were Mf positive, diagnosed by rapid test, and confirmed by TBF.

### Vector surveys

Vector surveys were done in all the target 11provinces in 47 IUs over 2018–2022 (see Table 3)*.* Vector surveys are conducted in 1% of sub-village where human blood surveys are conducted in that year. Over the 5 years, Mf was detected only in Narathiwat province in one isolate in 2018 (*Ma. annulata*/L2 stage) and 5 isolates in 2019 (*Ma. bonneae*/L1, L2 stage; *Ma. annulate*/L1, L2 stage; *Cq. crassipes*/L3 stage). All species were *B. malayi*. In the vector surveys, three nights of mosquito trapping were done. Mosquito species identification and Mf/larvae detection were done by ODPC laboratory and confirmed by PCR at the DVBD. Table 4 lists mosquito species collected and dissected in 11 provinces during vector surveys, 2018–-2022.

**Table 3:** List of mosquito species collected and dissected in 11 provinces during vector surveys, 2018–2022

Province	Mosquito species identified during vector surveys 2018–2022
Chiang Mai	*Culex pseudovisnui, Armigeres* spp., *Anopheles tesselatus, An. philipinesis, An. spendidus*
Lamphun	*Culex pseudovisnui*, *Armigeres* spp., *An. tesselatus*, *An. philipinesis*
Kanchanaburi	*Aedes desmotes*, *Ae. desmotes*, *Ae. albopictus*, *Ae. imitator*, *Ae. annandalei*, *Cx. quinquefasciatus*, *Ae. niveus*
Krabi	*Mansonia uniformis*, *Ma. dives*, *Ma. boneae*
Mae Hong Son	*Ae. albopictus*, *Ar. subalbatus*, *An. barbirostris*, *Ar. flavas*, *Ae. annandalei*, *Cx. quinquefasciatus*, *An. minimus*, *An. maculatus*, *An. sawadwongporni*, *Ae. poicilius*, *Ae. vitatus*
Nakhon Si Thammarat	*Ma. boneae*, *Ma. dives*, *Ma. uniformis*, *Cx. gelidus*
Narathiwat	*Ma. bonneae*, *Ma. annulata*, *Ma. indiana*, *Ar. subalbatus*, *Ae. albopictus*, *Anopheles* spp., *Coquillettidia nigrosinata*, *Cq. crassipes*, *Ma. uniformis*, *Cx. psuedosininsis*, *Ma. uniformis*, *Ma. annulifora*, *Cx. gelidus*, *Culex* spp.
Ranong	*Cx. quinquefasciatus*, *Cx. gelidus*, *Cx. sitiens*, *Ae. aegypti*, *Ae. albopictus*
Ratchaburi	*Ae. albopictus*, *Ae. subalbatus*, *Ma. indiana*, *An. barbirostris*, *Ae. vitatatus*
Surat Thani	*Ma. indiana*, *Ma. annulifera*, *An. karwari*, *An. letifer*, *Ae. albopictus*, *Culex* spp., *Ar. subalbatus*, *Ma. dives*, *Ma. bonneae*, *Ma. uniformis*
Tak	*Niveus* spp., *Ae. albopictus*, *Ae. imitator*, *Ar. subalbatus*, *Ar. leicesteria*, *Heizmannia* sp., *An. minimus*, *Ae. desmastes*, *Ae. annandalei*, *Ar. kesseli*, *Ae. poicilus*, *Cx. tritaeniorrynchus*

**Table 4:** Vector surveys: 2018–2022: 11 provinces: Number of IUs, mosquitoes dissected and positive with Mf and larva

Province	Total No. of IUs surveyed	2018		2019		2020		2021		2022	
		No. of mosquitoes dissected	No. of mosquitoes with Mf	No. of mosquitoes dissected	No. of mosquitoes with Mf	No. of mosquitoes dissected	No. of mosquitoes with Mf	No. of mosquitoes dissected	No. of mosquitoes with Mf	No. of mosquitoes dissected	No. of mosquitoes with Mf
Chiang Mai	1	0	0	0	0	0	0	29	0	0	0
Lamphun	1	0	0	0	0	0	0	0	0	63	0
Kanchanaburi	19	0	0	361	0	162	0	34	0	975	0
Krabi	1	0	0	0	0	0	0	0	0	26	0
Mae Hong Son	6	272	0	60	0	0	0	35	0	90	0
Nakhon Si Thammarat	2	26	0	0	0	0	0	0	0	30	0
Narathiwat	9	1132	1^a^	2344	5^b^	493	0	1036	0	514	0
Ranong	1	0	0	71	0	0	0	0	0	0	0
Ratchaburi	1	0	0	55	0	0	0	0	0	0	0
Surat Thani	2	108	0	0	0	0	0	0	0	12	0
Tak	4	231	0	381	0	0	0	0	0	0	0

^a^*Ma. Annulata*/ stage L2 (1 specimen)

^b^*Ma. bonneae*, stage L1,L2 (2 specimens), *Ma. annulata*, stage L1,L2 (1 specimen), *Cq. Crassipes*, stage L3 (1 specimen), *Ma. Annulata*, stage L2 (1 specimen) L = Larva stage, *IU* implementation unit

### LF chronic disease survey and management

**Fig. 4:**
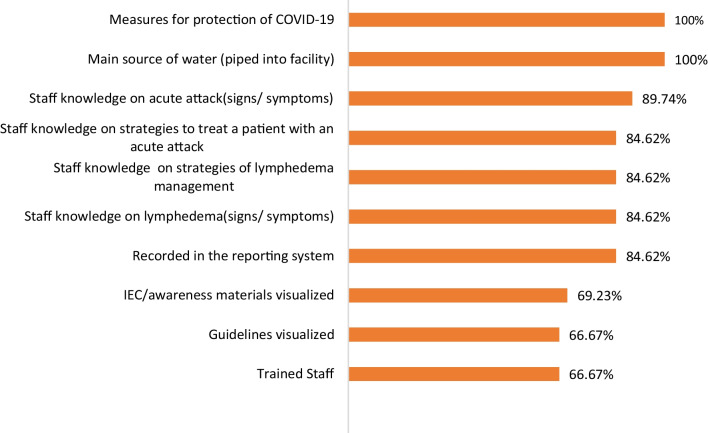
MMDP assessment sites: 2020: percentage (%) of health facilities compliant to MMDP indicators (*n* = 39). *MMDP* Morbidity Management and Disability Prevention, *IEC* Information, education, communication

In 2022, there were 85 LF cases in Thailand, which as of 2022 were under the care of 42 health facilities in 8 provinces (Table 5). The ODPC 11 Nakhon Si Thammarat monitors cases in the provinces of Chumphon, Surat Thani, and Nakhon Si Thammarat, while ODPC 12 Songkhla monitors cases in the provinces of Songkhla, Patthalung, Pattani and Narathiwat. An MMDP quality assessment was carried out by the DVBD and ODPC in 2020–-2021 in 7 provinces among health staff in 40 health facilities using the WHO Direct Inspection Protocol version 1.1 with 14 indicators with the addition of an indicator on COVID-19 (Fig. 4).

**Table 5:** LF patients on MMDP: 2018–2022

Provinces	2018				2019				2020				2021				2022			
	Mf+	L	E	Total	Mf+	L	E	Total	Mf+	L	E	Total	Mf+	L	E	Total	Mf+	L	E	Total
Chumpon	0	0	2	2	0	0	2	2	0	0	2	2	0	0	1	1	0	0	1	1
Surat Thani	0	0	11	11	0	0	9	9	0	0	9	9	0	0	8	8	0	0	8	8
Nakhon Sri Thammarat	0	0	65	65	0	0	52	52	0	0	42	42	0	0	41	41	0	0	38	38
Songkhla	0	0	0	0	0	0	0	0	0	0	1	1	0	0	1	1	0	0	1	1
Pattalung	0	0	2	2	0	0	2	2	0	0	2	2	0	0	2	2	0	0	2	2
Pattani	0	0	9	9	0	0	8	8	0	0	8	8	0	0	8	8	0	0	6	6
Narathiwat	13	0	12	25	17	7	12	36	21	0	15	36	13	7	15	35	8	6	13	27
Ratchaburi																	1	1	0	2
Total	13	0	101	114	17	7	85	109	21	0	79	100	13	7	76	96	9	7	69	85

Number of Mf positive, Lymphodema and Elephantiasis

*Mf+* microfilaria positive, *L* lymphedema, E* elephantiasis

The assessment in 2020, as part of the PVS, showed a reducing number of LF patients from 114 (99 with lymphoedema/elephantiasis) in 2017 to a total of 85 (76 with lymphoedema/elephantiasis) in 2022. Of the total patients in 2022, 38 (44.7%) were under the care of 13 health facilities in just one province of Nakhon Si Thammarat. Of the thirty-nine health staff surveyed, all knew of COVID-19 measures to be taken for prevention, 84% on average knew signs of symptoms of LF, lymphoedema, how to manage a case of LF, and report. Still, only 67% on average were trained and aware of guidelines and relevant IEC material for the management of LF.

### LF surveillance among migrants

Surveillance of migrant populations in Thailand for LF through various agencies are done as part of health screening required for migrant work permits or at ports of entry for both registered labour migrants [screening done by the PHO, Department of Medical Service—Bangkok Metropolitan Authority (BMA), One Stop Service (OSS), Office of the Permanent Secretary of MoPH] as well unregistered migrants (screening done by the Provincial office of Disease Control and Division of International Disease Control in Ports). Screening is done mainly by TBF or with FTS and depends on local arrangements with hospitals, laboratories, and available budgets. The total number of migrants screened by these agencies was 728,862 in 2018 but decreased to 611,485 in 2020 and 314,663 in 2021 due to the COVID-19 pandemic and movement restrictions. Data on the number of positives are not available to the DVBD. In the annual LF surveys among migrants conducted by DBVD and VBDU, (Table 6) FTS is used for screening, and a total of 12 antigen-positive cases among unregistered migrants were detected in 6 out of 10 provinces over 2018–2022. All positive cases were treated with DEC, followed up by local staff. No blood surveys were done in 2020 due to COVID-19 lockdowns and restrictions. In addition, in eight of these provinces where blood surveys were done, vector surveys were also conducted simultaneously, in the same area (Table 7). No vector surveys were done in 2020–2022 in migrant settlements due to COVID-19 lockdowns and restrictions. 2408 mosquitoes were collected over 2018–2022 and dissected by VBDC and ODPC, and no microfilaria/larvae were detected.

**Table 6:** Results of LF surveys among unregistered migrants: 2018–2022

Province	2018				2019				2021				2022		
	No. tested by FTS	Ag+	AGR (%)		No. tested by FTS	Ag+	AGR (%)		No. tested by FTS	Ag+	AGR (%)		No. tested by FTS	Ag+	AGR (%)
Bangkok	794	3	0.38		0	0	0		0	0	0		0	0	0
Tak	762	0	0		200	0	0		0	0	0		392	0	0
Samut Prakan	164	0	0		423	0	0		0	0	0		0	0	0
Chonburi	264	0	0		500	1	0.20		0	0	0		0	0	0
Ranong	498	0	0		0	0	0		339	0	0		0	0	0
Surat Thani	256	0	0		415	0	0		473	3	0.63		139	0	0
Chiang Mai	0	0	0		424	0	0		0	0	0		0	0	0
Nakhon Pathom	0	0	0		400	2	0.50		0	0	0		0	0	0
Phuket	0	0	0		484	2	0.41		0	0	0		167	0	0
Chumphorn	0	0	0		507	1	0.20		32	0	0		0	0	0
Total	2738	3		0	3353	6		0	844	3		0	698	0	0

2020—No blood surveys conducted among migrants due to COVID-19 lockdowns and restrictions

*AGR* antigen rate, *LF* lymphatic filariasis, *FTS* filaria antigen test strip

**Table 7:** Vector surveys in migrant settlements: 2018–2019

Province	No. of districts	No. of sub-districts	No. of village	2018			2019		
				Mosquito Species (number collected)	Total No. of mosquitoes collected	Mosquitoes Microfilaria Positive	Mosquito Species (number collected)	Total No. of mosquitoes collected	Mosquitoes Microfilaria Positive
Tak	1	1	1				*Cx. quinquefasciatus (271), Cx. gelidus (1), Cx. vishnuis (1), Ma. uniformis (5), Aedes aegypti (1), Ar. subalbatus (17)*	296	0
Samut Prakan	NA			*Culex gelidus (30), Cx. quinquefasciatus (103), Cx. vishnuai (2), Ma. aunalifera (79), Mansonia uniformis (15), Ma. bonnea (5), An. barbumbrosus (4), An. umbrosus (3)*	241	0	*Culex* spp. (36)	36	0
Chonburi	NA			*Annulata barbirostris (2), Armigeres* spp.* (3), Cx. quinquefasciatus (86), Cx. fuscocephala (1), Cx. bitaeniorhychus (92), Cx. Gelidus (7), Ma. uniformis (14), Ma. aunulifera (2)*	207	0	*Culex* spp.	33	0
Ranong	1	1	1	*Cx. quinquefasciatus (350), Cx. selidus (4), Cx. sitiens (170), Ae. aegypti (17), Ae. albopictus (6)*	547	0	*Cx. quinquefastus (10), Cx. gelidus (6), Cx. sitiens (82), Ae. aegyti (6), Ae. albopictus (13), Ar. subalbatus (132)*	249	0
Surat Thani	2	2	3	*Ma. uniformis (8), Ma. indiana (13), Ae. albopictus (1), Cx. quinquefasciatus (2), Culex* spp*. (31), Ar. subalbatus (12)*	67	0	*Cx. quinquefasciatus (24), Cx. vishui (140), Cx. whitei (100), Ma. uniformis (11), Ar. subalbatus (8)*	283	0
Chiang Mai	1	1	1				*Cx. quinquefasciatus (160), Ar. subalbatus (46), Cx. pseudovishnui (30), Ma. indiana (16), Ma. uniformis (14), Ae. aegypti (2)*	268	0
Phuket	2	2	2				*Culex* spp.*(5), Ae. albopictus (8), Culex* spp*.(3), Ae. albopictus (2), Ae. aegypti (5)*	23	0
Chumphon	1	1	1				*Cx. quinquefasciatus (129), Cx. sitien (16), Ae. aegypti (9), An. epiroticus (4)*	158	0

2020–22 No vector survey were done in migrant settlements due to COVID-19 lockdowns

NA: not applicable

Tak province borders to Kayin state in Myanmar. Two out of the five townships in Myanmar are endemic for LF. MDA for LF has been stopped in these two townships, and TAS-1 has been conducted with TAS-2 planned for 2023.

The provinces of Ratchaburi, Petchaburi, Chumporn, Prachuapkirikhan, and Ranong border to Tanintharyi region in Myanmar. Three of the four townships in this region are endemic for LF, of which two have stopped MDA for LF, conducted TAS-1 and TAS-2 planned for 2023.

In the Kayah and Shan regions of Myanmar that border to Thailand, LF is not endemic.

### Lymphatic filariasis (LF) in DP camps

Data on LF surveys and cases are not available prior to 2018. A prior survey in one camp in Tak province in 2018 tested 2634 persons using thick blood film (TBF) and found 20 positive cases (microfilaria positive rate = 0.76%) who were treated after the survey in addition to mass during administration (MDA) in the camp. In 2019, LF surveys were conducted in 4 camps. The screening was based on available financial, human resources, and availability of FTS. 5178 persons were tested, and 10 antigen-positive cases were detected (average AGR: 0.27, range: 0–0.29). All cases were treated. In 2022, due to the COVID-19 pandemic and available resources, LF surveys were only done in one DP camp, with 1283 (55%) of the camp population tested by FTS and 5 antigen-positive cases (AGR: 0.39), Table 8. Vector surveys to identify the presence of microfilaria are not done in DP camps. Cases detected in the DP camps are not included in the annual number of cases reported by the DVBD.

**Table 8:** Results of LF human blood surveys in displaced population (DP) camps in Thailand: PVS 2019 and 2022

No	Province	Number of DP camps	2019				2022			
			DP camp population in 2019	No. of persons tested	Ag+	AGR (%)	DP camp population in 2022	No. of persons tested	Ag+	AGR (%)
1	Tak	3	58,807	751	0	0	NA	0	0	0
2	Mae Hong Son	4	31,711	1480	4	0.25	NA	0	0	0
3	Kanchanaburi	1	2760	339	1	0.29	2325	1283	5	0.39
4	Ratchaburi	1	5342	377	1	0.27	NA	0	0	0
	Total	9	98,620	2947	6		2325	1283	5	0.39

*DP* displaced population, *Ag+* antigen positive, *AGR* antigen rate, *NA* data not available

### LF in cats

In 2018, the microfilaria rate among cats in Narathiwat province was 1.9% and in Surat Thani 18.6%, Table 9. It is unclear why there was a high rate for only 1 year in Surat Thani. However, the rates declined in subsequent years with no Mf positive cats detected in Surat Thani and a microfilaria rate of 0.7% in Narathiwat. All *Brugia* species are confirmed by PCR, at DVBD, and for 2018–2022 all were *B.malayi*. A register of household cats is maintained by ODPC 11 and the VBDC. The register records the number and names of cats in each house For Narathiwat by the Phikulthong Royal Development Study Center in Narathiwat with the name/characteristics of the domestic cat and record of blood testing and ivermectin injection at a dose of 0.1 cc/kg delivered subcutaneously. In IUs where Mf positive cats were detected, new human Mf cases were also detected (2019: 1; 2021: 1; 2022: 1) (Table 10).

**Table 9:** Number of cat survey, and microfilaria positive rate

Province	No. of cats tested_2018	No. of Mf+	Mf rate (%)	No. of cats tested_2019	No. of Mf+	Mf rate (%)	No. of cats tested_2020	No. of Mf+	Mf rate (%)	No. of cats tested_2021	No. of Mf+	Mf rate (%)	No. of cats tested_2022	No. of Mf+	Mf rate (%)
Krabi	0	0	0	48	0	0	64	0	0	71	0	0			
Nakhon Si Thammarat	0	0	0	46	0	0	55	0	0	67	0	0			
Surat Thani	43	8	18.60	61	0	0	0	0	0	68	0	0			
Narathiwat	211	4	1.90	818	9	1.1	451	3	0.67	601	2	0.00	573	4	0.70
Total	254	12	4.72	973	0	0	570	3	0.53	807	2	0.25	573	4	0.70

*Mf+* microfilaria positive

**Table 10:** IUs with both human and cat Mf + : Mf and Mf rate: 2018–2022

Year^a^	Total no. IUs with human blood survey	IUs with both human and cat Mf+	Human blood survey				Cat blood survey			
			Target Population	TBF	Mf+	Mf rate (%)	Target Population	TBF	Mf+	Mf rate (%)
2019	10	3	885	884	8	0.9	187	158	8	5.1
2021	8	1	248	239	1	0.4	34	29	1	3.4
2022	11	1	377	374	2	0.5	70	61	1	1.6

*TBF* thick blood film, *Mf+* microfilaria positive, *IU* Intervention/implementation unit

^a^There were no IUs recorded with both human and cat Mf+ in 2018 and 2020

## Discussion

### Human blood surveys

The human blood surveys over 2018–2022 showed that 144 IUs (40.2%) were surveyed (a total of 357 IUs), with a median population coverage of 88.7% among the surveyed areas. The Thailand LF PVS strategy for human blood surveys is to study 10% of total IUs per year for 10 years. Hence, at the 5-year mark (5 years: 2018–2022), the achievement is slightly below the targeted 50% total IU coverage but a significant achievement as most of the eleven provinces continued to conduct human blood surveys in designated IUs over 2020–2022 despite the COVID-19 pandemic. There were geographical differences in coverage, mainly due to COVID-19 border restrictions on travel, access to villages under lockdown measures, and COVID-19 varying case burden and transmission patterns. Over 2020–2022, different provinces were affected by the sequential waves of the COVID-19 pandemic. In provinces along the border with Myanmar, these affected both in-outbound migrants and also for health staff access to communities, in particular in 2022 due to the political situation in the neighboring country. In provinces of Chiang Mai, Ranong, Krabi, and Nakhon Si Thamamarat, where there are three or fewer IUs in each of these provinces, PVS human blood surveys could be integrated with other community health efforts and programs as a more cost-effective strategy. The role of PCD through routine notifiable disease surveillance is essential in the PVS phase in both endemic provinces and in provinces where there is a high risk of importation, especially among migrants, as recorded in 2022 in the province of Ratchaburi. The reasons for ongoing transmission in Narathiwat could be attributed to the persistence of vectors in and around a protected peat swamp forest of 66,000 acres, which spreads over 3 districts of Tak Bai, Sungai Kolok, and Sungai Padi, where the majority of the LF cases in Narathiwat are reported from. The persistence of non-modifiable factors (where source reduction is not possible) favourable for vector breeding, in this case, protected peat swamp forests, and a few IUs with zoonotic *B.malayi*, will result in incident cases of LF occurring over the PVS phase. Aiming for zero cases will be extremely difficult, even if MDA were to be resumed. Other preventive measures, ie prevention of mosquito bites, could be considered, although the 3 districts are currently free of malaria and dengue. More research is needed in these IUs for the effectiveness and acceptability of insecticide-treated bed nets, and mosquito repellents taking into account the biting behaviour and exposure to the main vector, *Mansonia* spp. In addition, in IUs that document local infection with Mf rate > 1%, triple therapy or IDA (ivermectin, DEC, and albendazole) could be considered as part of Thailand’s PVS strategy to address focal persistence of LF [11, 12].

### Vector surveys

In Thailand and other Southeast Asian countries, the principal vectors of *B. malayi* are *Mansonia* mosquitoes, including *Ma. uniformis, Ma. indiana, Ma.annulifera, and Ma. onneae* [13, 14]. With the current program protocol of conducting vector surveys in 1% of IUs in previously endemic provinces, only Narathiwat Province has detected filarial larvae in vectors. These larvae found in vectors were not found in IUs (sub-village) where MF human cases were detected but in the same subdistricts. Only Paye IU in Narathiwat province has demonstrated possible autochthonous transmission. As vector surveys and mosquito dissection is a labor-intensive activity, and transmission of lymphatic filariasis is significantly influenced by vector density [15, 16], the current protocol may need revision in light of the low Mf/filarial larvae detection rate. Intervention programs rolled out in endemic areas should be specific and targeted in each endemic foci where transmission is focal [17, 18]. At the same time, the integration of vector surveys with other diseases, for example malaria or dengue, could be considered where both human and financial resources are limited. In addition, given the low transmission levels in most endemic communities in Thailand, molecular-based techniques, as shown in other countries, may be an effective tool for xenomonitoring [19, 20].

### LF chronic disease survey and management

The last assessment of the quality of lymphedema services in 2017, before validation of elimination, identified high staff turnover that affected the knowledge of responsible officers and the availability of MMDP materials. The assessment in 2020 also identified issues with the retention of health staff who were previously trained, either due to being repositioned to other health offices and tasks or retirement. Subsequently, their replacements were not trained in MMDP management. In most health facilities, the follow-up home visits of patients were done by the village health volunteers who integrated with other home visit health activities. The assessment also highlighted improvements needed for the MMDP. These included integrating MMDP care as part of the long-term care of the elderly, providing MMDP guidelines in digital media format, which can consist of knowledge on LF and MMDP in two languages, Thai and Melayu/Jawi languages as well as for staff training purposes for example, demonstrations of caring for patients with filariasis symptoms. In 2022, DVBD initiated integrated VBD TOT training courses for regional offices—epidemiology, surveillance, sample collection and vector management. The DVBD is developing online training modules to ensure regular training of health staff involved in MMDP. There is also a need for producing more brochures/posters about filariasis diseases and that these public health facilities have antifungal drugs and primary care equipment boxes. The following survey is planned for 2023.

### LF surveillance among migrants

Although the number of migrants tested, method, and target group through various agencies are reported, the figures are for general health checkups, and inclusion of TBF is variable depending on local arrangements with hospitals and laboratories and available budget. In addition, the number of LF positives from these migrant health screenings are not captured in the reporting. However, before COVID-19, over 2018–2019, five nonendemic provinces including Bangkok, reported twelve cases in the LF migrant screening program (provincial AGR: 0.2–0.5%), where all cases were among Myanmar nationals. In 2021, three cases among migrants were reported in the endemic province of Surat Thani (AGR 0.63%). Although these screening programs showed low yield over the 2018–2022 PVS period, these were perceived as essential to continue by the respective provinces, with more than 5 provinces conducting these screenings annually over 2018–2019. Due to the COVID-19 restrictions, this activity was not implemented in 2020 but resumed in 2021 and 2022. Due to budgetary constraints, only 4 provinces conducted this in 2021 and 2022, respectively, against the PVS target of five provinces per year. Both measures, the MDAs conducted in Myanmar [vide supra] and the screening and treatment of migrants in Thailand, were probable contributors to the decline in the number of LF cases detected in Thailand among Myanmar migrants. Among Thailand’s border with its neighbours, only Myanmar has several provinces that are endemic for LF caused by *W. bancrofti* and transmitted mainly by *Culex quinquefasciatus*. Although there has been some debate on human-vector combinations on the risk of *W. bancrofti* transmission across the Thai-Myanmar borders [21, 22], current data/information thus far is not sufficient to understand the vulnerabilities on how contagious the parasite is in such complex epidemiological settings as well as the receptivity of the vector in different ecological settings along the borders [3, 24]. In vector surveys in migrant settlements a total of 2408 mosquitoes were collected and dissected over 2018–2019, and no microfilaria or larva was detected, likely suggesting that the positive MF cases among migrants were importation and not indigenous transmission.

Since 2001, the Thai MoPH set up the migrant health insurance scheme for all migrants (documented and undocumented) who are not covered by social health insurance, allowing mandatory health screening (during the first entry and subsequent yearly renewal of the residence permit) which includes testing for *bancrofti*an Mf (Mf provocation test with DEC) which is done at all district hospitals and for which a full course of treatment (single dose of DEC + ALB) is offered if found to be positive. In addition, the local health facilities are encouraged to treat the immigrant population regardless of legal status. Barriers to receiving DEC were lack of official documents, unemployed status, daily employment, short-term immigrant status, and living in a fishery area for immigrants [23].

DVBD needs increased collaboration with the appropriate agencies (vide supra) to obtain testing and outcomes to map distribution better and monitor migrant patient follow-up. Better profiling migrant populations [24] and developing criteria for prioritized group/s for periodic surveillance could be used to detect any LF cluster that may arise promptly [25, 26].

In the 9 displaced population (DP) camps located in 4 provinces in Thailand along the border with Myanmar, human blood surveys for LF are dependent on the availability of resources annually both at DVBD and in the PHO. Although surveys conducted in 2019 and 2022 have been limited in coverage of testing and yield of positive cases, a prior survey in one camp in Tak province in 2018 tested 2,634 persons using TBF and found 20 positive cases (microfilaria positive rate: 0.76%) who were treated after the survey in addition to MDA in the camp (personal communication, SR). Since there have been no records of lymphoedema or elephantiasis cases in DP camps, MMDP has not been initiated in these camps. Should there be a need, health clinics in the DP camps managed by an international NGO will be engaged to provide MMDP services. Given the current unrest in Myanmar and the increased movement of population across the border into Thailand, the current PVS strategy will require additional resources to extend both human blood and vector surveys in selected camps where previous cases have been recorded and that report an influx of new DPs into the camps. An appropriate sampling methodology will need to be developed for human blood surveys in addition to MDA protocols. Where positive LF is found, in addition to treatment, MMDP needs to be initiated by the NGO responsible.

### LF in cats

As early as the late 1980s, cat surveys documented *B. malayi* and *B. pahangi* infection among domestic cats in all four *B. malayi* endemic provinces of Surat Thani, Nakhon Si Thamarat, Krabi, and Narathiwat. [5, 13]. Beginning in 2003, active surveillance of cats in areas with > 1.0% Mf rate among cats was done along with mass treatment of cats with ivermectin given subcutaneously as a strategy to interrupt possible zoonotic transmission. PVS results (10% of previously reported *B. malayi* IUs) over 2018–2022 showed positive Mf in cats in some IU where human Mf cases were found. More definitive studies are needed, although current cat surveys and treatment could be justified in targeted IUs in Narathiwat, where new human cases are found.

### LF and zoonotic transmission

The occurrence of other species of filarial parasites, such as *B. pahangi* and *Setaria* spp., have been documented in Thailand [27] and demonstrated *Ar. subalbatus* to be a vector of zoonotic *B. pahangi* in Suratthani, Southern Thailand, where Thai children have been infected with zoonotic *B. pahangi*. Four cases were documented over 2012–-2020 in children less than 2 years of age living in rubber and oil palm plantations with varying manifestations of fever with or without lymphatic pathology. In two cases, proximity to *B. pahangi* nfected dogs or cats was documented. Microfilaria of *Setaria* spp. were also found in bullfighting cattle in the southern part of Thailand [28]. Currently, *B. pahangi* can be observed in *Ar. Subalbatus*, found in abundance in rubber or oil palm plantations, is the natural vector for zoonotic *B. pahangi* and can also transmit the disease to humans [29] through reservoir animals such as cats and dogs [30]. Although genetically, *B. pahangi* and *B.malayi* are closely related, their physiology, vector competence, and transmission potential differ [27]. As the ecological landscape in southern Thailand changes with peri-urban development, understanding both the exposure and receptivity of human-vector-animal interactions will be necessary [31]. This will require strengthening the capacity in diagnosis and surveillance for zoonotic infections through a One Health approach. The Phikulthong Royal Development Study Center in Narathiwat continues monitoring for zoonotic LF transmission while focusing on soil-transmitted helminthiasis (STH) and leprosy control as well. It could be an essential institution to take this forward.

Our study also has limitations. Only testing data on migrant worker routine health screening through various agencies was available to the DVBD. The yield from these screenings was not available to the authors. Further analysis is required to determine the magnitude of imported cases of LF. Our study could not assess the impact of health education activities and tools for LF prevention and control in the community. This would be an important area for further research for better targeting of communities at risk given the low prevalence even in persisting endemic IUs.

## Conclusions

Sustained commitment by the government and dedicated health staff on the ground throughout the elimination phase not only ensured the NPELF objectives were finally met in 2017 but also in ensuring that high quality of care is continued for chronic LF patients and adoption of a robust PVS program.

In 2022, after 5 years of PVS, Thailand has re-surveyed 41% of all its 357 previously endemic IUs in 11 provinces and demonstrated ongoing transmission in only one province of Narathiwat, where Mf prevalence of *B. malayi* remains below the current WHO transmission threshold of 1%. The WHO emphasizes that validation of elimination as a public health problem implies a potentially reversible state, and countries should continue to conduct PVS. While guidance from WHO defining criteria/s to verify the elimination of transmission is yet to be determined, it is envisaged Thailand will recover from program setbacks in 2020–2021 due to COVID-19 and achieve 100% coverage of its PVS surveys in its IUs by 2027. This includes strengthening passive surveillance nationwide, targeted migrant screening in specified provinces and DP camps, innovative methods for MMDP refresher training, especially for newly stationed health facility staff, and ensuring integration of MMDP into sub-district health services. In Narathiwat province, for IUs with > 1% Mf rate, IDA-based intervention could be adopted as an accelerated approach as part of Thailand’s PVS strategy.

## Data Availability

The datasets are available from the corresponding author upon reasonable request.

## Abbreviations

Ab: Antibody
Ag: Antigen/antigenaemia
Ag+: Antigen positive
AGR: Antigen positive rate
ALB: Albendazole
*Ar.*: *Armigeres*
BMA: Bangkok Metropolitan Authority
COVID-19: Coronavirus disease 2019
DVBD: Division of Vector Borne Diseases
DDC: Department of Disease Control
DEC: Diethylcarbamazine
DP: Displaced populations
EU: Evaluation unit
FTS: Filaria antigen test strip
GPELF: Global Programme to Eliminate Lymphatic Filariasis
IDA: Ivermectin-DEC-albendazole
IEC: Information, education, communication
IU: Intervention/implementation unit
LF: Lymphatic filariasis
M&E: Monitoring and evaluation
Mf+: Microfilaria positive
MDA: Mass Drug Administration
MMDP: Morbidity Management and Disability Prevention
Mf: Microfilaria
MoPH: Ministry of Public Health
NGO: Non-Governmental Organization
NTDs: Neglected tropical diseases
NPELF: National Programme to Eliminate LF
ODPC: Regional Office of Disease Control and Prevention
PCD: Passive case detection
PCR: Polymerase chain reaction
PHO: Provincial Health Office
PVS: Post-validation surveillance
RDT: Rapid diagnostic test
SEARO: WHO South-east Asia Regional Office
STH: Soil-transmitted helminthiases
TAS: Transmission assessment survey
TBF: Thick blood film
VBDC: Vector Borne Disease Centre
VBDU: Vector Borne Disease Unit
WHO: World Health Organization
